# Synthesis and Characterization with Antineoplastic, Biochemical, Cytotoxic, and Antimicrobial Studies of Schiff Base Cu(II) Ion Complexes

**DOI:** 10.1155/2015/923087

**Published:** 2015-07-29

**Authors:** M. M. Haque, Md. Kudrat-E-Zahan, Laila Arjuman Banu, Md. Shariful Islam, M. S. Islam

**Affiliations:** Inorganic Research Laboratory, Department of Chemistry, University of Rajshahi, Rajshahi 6205, Bangladesh

## Abstract

Copper(II) complexes containing two Schiff base ligands derived from 2-hydroxybenzaldehyde with 2-aminophenol and 3-aminophenol have been synthesized and characterized by means of analytical, magnetic, and spectroscopic methods. Bacteria, fungus, *Entamoeba histolytica*, and antineoplastic activities of the synthesized complexes have been determined by monitoring the parameters cell growth inhibition, survival time of tumour mice, time-body relation, causing of intraperitoneal cells and macrophages, alkaline phosphatase activity, hematological effect, and biopsy of tumour.

## 1. Introduction

Schiff base metal complexes based research works have been widely carried out from 1930, because of their biological and industrial applications [1–5]. The use of metal complexes as pharmaceuticals has shown promise in recent years particularly as anticancer agents [6]. Previously, synthesis and properties of thiocyanato complex of low valent metal ions containing different monodentate auxiliary ligands have been reported from our laboratory [7–9]. Worldwide spread of drug-resistant bacteria is now a critical problem in global health. To find new drug, recently, we studied few mixed-ligand complexes containing heterocyclic amine as secondary ligands and few Schiff base containing complexes [10–12]. In present study, mixed-ligand complexes of Cu(II) containing the Schiff base ligand derived from 2-hydroxybenzaldehyde with 2-aminophenol/3-aminophenol and bidentate auxiliary ligands were synthesized and characterized. The auxiliary ligands used were potassium thiocyanato, 2-aminopyridine, and 2-phenylenediamine. Antineoplastic, biochemical, cytotoxic, and antimicrobial activities of the complexes were also studied.

## 2. Experimental

### 2.1. Physical Measurement

The weighing operation was performed on a METTLER PM-200 electronic balance. Conductivity measurements were carried out in dimethyl sulfoxide (DMSO) using a WPACMS 35 conductivity meter and dip-cell with platinized electrodes. The melting or decomposition temperatures of all the prepared metal complexes were observed in an electrothermal melting point apparatus model number AZ6512. The SHERWOOD SCIENTIFIC Magnetic Susceptibility Balance was used for the present investigation. Infrared spectra as KBr disc were recorded in a SIMADZU FTIR-8400 (Japan) infrared spectrophotometer, from 4000 to 400 cm^−1^. The absorbances of the complexes were recorded on SHIMUDZU Spectrophotometer. Analyses of the complexes for carbon, hydrogen, and nitrogen were carried out by Microanalytical Services at the University of St. Andrews, Scotland. Transplantable tumour (Ehrlich's Ascites Carcinoma, EAC) used in this research was obtained from Indian Institute of Chemical Biology (IICB), Calcutta, India. In vivo antineoplastic activity of the test complexes was determined by measuring the effect of the test complexes on tumour cell growth inhibition, survival time of tumour bearing mice, hematological parameters, and serum alkaline phosphatase activity of tumour bearing mice. Tumour growth was monitored by recording daily weight change. The concentration of haemoglobin was measured by the usual procedure using Sahli's haemometer.

### 2.2. Procedure of Preparation of Schiff Base (SB-1)

**Figure 1:**
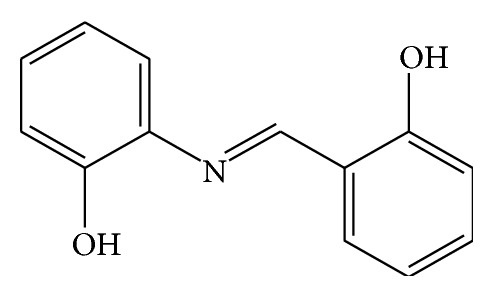
(2-Hydroxy-benzylidene)-(2-hydroxy-phenyl)-amine (SB-1). Yield: 3.0 g (78%). Anal. Calc. (%): C, 73.2; H, 5.2; N, 6.6. Found: C, 73.1; H, 5.1; N, 6.7. M.p. 160–162°C.

The Schiff bases were prepared by the condensation of 2-hydroxybenzaldehyde with 2-aminophenol. 2-Hydroxybenzaldehyde (1.7 g, 0.014 mol) in absolute ethanol (20 mL) was added to an ethanolic (30 mL) solution of 2-aminophenol (1.5 g, 0.014 mol). The mixture was heated to reduce the volume to 25 mL, and then it was cooled in an ice-bath. The black crystalline product was isolated and washed with hot ethanol. The structure of SB-1 is shown in Figure 1.

#### 2.2.1. Procedure of Preparation of Test Compound K[Cu(SB-1)(SCN)], SB-1 = C_13_H_9_NO_2_

An appropriate solution of CuCl_2_·H_2_O (0.005 mol) in absolute ethanol (25 mL) was added to an ethanolic (30 mL) solution of potassium thiocyanate (0.005 mol). The solution was filtered and the filtrate was added to the methanolic solution of C_13_H_9_NO_2_H_2_ (SB-1) (0.005 mol, 80 mL). The resulting mixture was boiled on a water bath for 5 minutes and cooled. The complexes were separated, washed with hot ethanol, and dried in vacuo over P_4_O_10_.

### 2.3. Procedure of Preparation of Schiff Base (SB-2)

**Figure 2:**
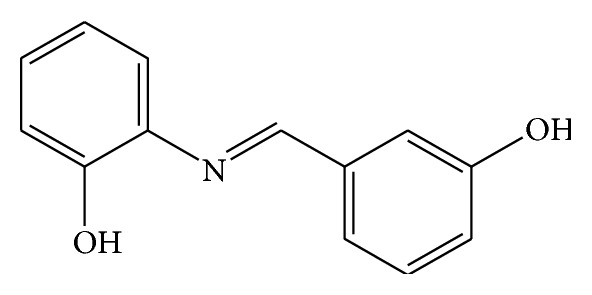
(3-Hydroxy-benzylidene)-(2-hydroxy-phenyl)-amine (SB-2). Yield: 3.1 g (82%). Anal. Calc. (%): C, 73.2; H, 5.2; N, 6.6. Found: C, 73.0; H, 5.2; N, 6.5. M.p. 160–162°C.

The Schiff base was prepared by the condensation of 2-hydroxybenzaldehyde with 3-aminophenol. 2-Hydroxybenzaldehyde (1.7 g, 0.014 mol) in absolute methanol (20 mL) was added to an ethanolic solution (30 mL) of 3-aminophenol (1.5 g, 0.014 mol). The mixture was heated to reduce the volume to 25 mL and then it was cooled in an ice-bath. The black crystalline product was filtered and washed with hot ethanol. The structure of SB-2 is shown in Figure 2.

#### 2.3.1. Procedure of Preparation for [Cu(SB-2)(NN)] [NN = 2-Aminopyridine/2-Phenylenediamine]

25 mL of an ethanolic solution of the metal chloride (0.005 mol) [CuCl_2_·H_2_O] was added to 30 mL of an ethanolic solution of the above prepared Schiff base (1.05 g, 0.005 mol). Then 20 mL of an ethanolic solution of [NN] (0.005 mol) was added to the metal salt-Schiff base solution. The resulting mixture was boiled on a water bath for 5 minutes and after that it was cooled. The complexes that were separated were washed with hot ethanol and dried in vacuo over P_4_O_10_.

## 3. Results and Discussion

### 3.1. Elemental Analysis and Conductivity Measurement

The analytical data and physical properties of the synthesized complexes are given in Table 1. The molar conductances in DMSO indicate that the complexes are all 1 : 1 electrolytes [13, 14].

### 3.2. IR Studies

*Complex A*. The infrared spectral data are shown in Table 2. The Schiff base C_13_H_9_NO_2_H_2_ [SB-1] behaves as tridentate dinegative ligand coordinating at the imino nitrogen and two oxygen atoms. In the complexes, the shift of *ν*(C=N) mode in a frequency 1605 cm^−1^ relative to the free ligand value 1610–1620 cm^−1^ (for ligand C_13_H_9_NO_2_) indicates that bond formation takes place through the imino nitrogen atom. The *ν*(OH) band observed in the free Schiff base (SB-1) disappears upon coordination, which indicates deprotonation and coordination at the oxygen sites. Furthermore, the presence of *ν*(M-O) and *ν*(M-N) linkages of bands at 455–535 cm^−1^, respectively, was observed for all the complexes (A) [15–17]. The ambidentate thiocyanate ligand can give either M-NCS or M-SCN bonding sequence, which nevertheless reveals the acidity of the metal ions. The complexes also display *ν*(CN) at 2100 cm^−1^ characteristic of S-bonded thiocyanato moieties. In Pearson's terminology, these are soft acids. The *ν*(CS) modes appear at lower frequencies in the M-S-C=N complexes than those in the M-N-C=S complexes [8, 9, 18]. The band of *ν*(CS) at 750 cm^−1^ is characteristic of M-S-C=N bonding sequence. This is further apparent from the *ν*(M-S) modes at 350 cm^−1^ in the far infrared spectra of the complexes.

*Compounds B and C*. The free Schiff base ligand shows characteristic bands at 3530 cm^−1^ for *ν*(OH) and 1607 cm^−1^ for *ν*(C=N). In the complexes, *ν*(C=N) of the Schiff base ligand remains practically unchanged showing that the imino nitrogen does not participate in bonding. The IR spectrum of free 2-phenylenediamine shows *ν*(NH_2_) modes at 3400 and 3378 cm^−1^, respectively. These bands are also shifted to lower frequencies in the complexes (B, C) (3315, 3232 cm^−1^) indicating coordination by the amino nitrogen but appear at 3290 cm^−1^ and 3150 cm^−1^ in compound B. This is also evident from the appearance of bands at 285–310 cm^−1^ which are tentatively attributed to the *ν*(M-N) mode. Further, in complexes with 2-aminopyridine (B), the *ν*(C=N) mode appears at 1555 cm^−1^ indicating that the ring nitrogen is coordinated to the metal atom.

### 3.3. Magnetic Moment and Electronic Spectra

The effective magnetic moments and electronic spectral components are shown in Tables 1 and 3. All the complexes are paramagnetic and show magnetic moment between 1.90 and 2.00 B.M corresponding to one unpaired electron. In electronic spectra three bands were observed at around 15455, 19500, and 22172 cm^−1^ corresponding to the transitions, ^2^B_1g_ to ^2^A_1g_, ^2^B_1g_ to ^2^E_g_, and charge transfer, respectively. These bands are consistent with square planar geometry [19].

## 4. Antineoplastic Activity of the Test Compounds

### 4.1. The Effect of Test Compounds and Bleomycin on Ehrlich Ascites Carcinoma (EAC) Cell Growth Inhibition

Treatment with test compounds resulting in maximum cell growth inhibition on compounds A, B, and C as evident from 95.21%, 87.40%, and 89.49%, reduction of tumour cell, which was found to be equivalent to standard or nearly standard antitumour agent bleomycin, which shows that cell growth inhibition is 94.90%. The results are shown in Table 4.

### 4.2. Effect of Test Compounds on Survival Time of EAC Cell Bearing Mice

It was found that treatment of tumour induced test animals with the compounds A, B, and C resulting in the increase of life span 35.70%, 16.98%, and 22.32%, respectively, when compared to control mice (life span 21.37 days). It was noticed that the anticancer antibiotic bleomycin increased the life span by 87.3% when compared to control. The results are shown in Tables 5 and 6.

### 4.3. Effect of Test Compounds and Bleomycin on Average Tumour Weight

Treatment of the test animals with test compounds, previously incubated with EAC cells, resulted in the inhibition of tumour growth. In case of treated group the body weight was growing slowly and by 53.99%, 46.78%, and 51.46% less in compounds A, B, and C, respectively, compared to control group after 20 days of tumour inoculation. But in case of bleomycin, this value is 52.63% compared to control group. DMSO does not show any change of body weight compared to control group.

### 4.4. The Effect of Test Compounds on Hematological Parameters in Normal and Tumour Bearing Mice

Hematological parameters were studied in normal and tumour bearing mice. All were treated with test compounds for 12 days of tumour transplantation and after 12 days they were sacrificed and blood was collected for hematological examination. Number of mice were four. Results were shown in mean ± SEM and compared with normal (without EAC bearing mice) and control (EAC bearing mice) group as shown in Table 7. The growth of tumour in mice induced by EAC cells effect in acute anemic condition as indicated by the significant decrease of the Hb% when compared to normal test animals under similar condition on day 12. The total white blood cell (WBC) count was also markedly decreased in the control group. In differential count of WBC, lymphocyte count was also found to be decreased and neutrophil count was increased on day 12 of tumour inoculation but no significant changes were observed in monocyte count on day 12 of the tumour inoculation as compared with normal mice. Effect of test compounds on hematological parameters of normal mice was also determined. No significant effect was found.

### 4.5. The Effect of Test Compounds on Serum Alkaline Phosphate Activity

Serum alkaline phosphatase activities were studied in normal and tumour bearing mice. Tumour bearing mice were treated with test compounds for 5 days of tumour transplantation and after 5 days they were sacrificed and blood was collected for determination of serum phosphatase. Serum alkaline phosphatase activity level in tumour bearing was decreased due to tumorigenesis when compared to the normal. Treatment with the test compounds restores the enzyme activity towards normal significantly. The serum alkaline phosphatase activity of the test compounds is shown in Table 8.

### 4.6. Effect of Test Compounds on the Enhancement of Peritoneal Cells and Macrophages of Life

Treatment with the test compounds did not show any effect on the enhancement of number of peritoneal cells but the number of macrophages increased (Table 9).

### 4.7. The Effect of Test Compounds on Generation of MDA by Lipid Peroxidation in Serum of Normal Mice

Animals were treated with test compounds for 4 consecutive days. Sera from mice were collected on day 5 and malondialdehyde (MDA) concentration was measured. The dose of the test compounds A, B, and C was 8 mg/kg, 108 mg/kg, and 168 mg/kg, respectively. Effect of test compounds on normal mice showed that there was markedly increase in MDA, which indicated that there was release for free radical. The obtained data are shown in Table 10.

### 4.8. Histopathological Effect of Test Compounds

Ehrlich Ascites Carcinoma (EAC) cell inducing tumour at the site of injection was very prominent and showed fast growth, increased in size, and bulged out in skin. The histological feature shows necrosis at the centre and viable growing cells in the periphery. Some inflammatory reactions lymphocytic in nature with reduction of hair follicle were observed. The number of mitosis was observed which increases greatly. When treated with test compounds and bleomycin growth rate of tumour is reduced, inflammatory reaction has also increased to some extent (Table 11). Necrotic area is increased and hair follicles show their normal appearance.

### 4.9. Effect of Test Compounds on Total Protein in Peritoneal Fluid

Inoculation of Ehrlich Ascites Carcinoma (EAC) cell in peritoneal cavity causes accumulation of fluid, which is rich in protein. But when treated with test compounds, the protein present in the peritoneal fluid is reduced and fluids accumulate in the peritoneal cavity very slowly (Table 12).

## 5. Antifungal Activity of the Test Compounds

### 5.1. Zone of Inhibition of Antifungal Activity of Test Complexes

The antifungal activity of the test complexes against different fungi was investigated by using the doses of 80 *μ*g/disc, where standard antibiotic disc of Nystatin (45 *μ*g/disc) was used for comparison purpose. The diameter was evaluated 4 mm, 2 mm, and 3 mm against* tinea pedis*; 8 mm, 23 mm, and 10 mm against* Aspergillus niger*; 22 mm, 6 mm, and 8 mm against* Coniothyrium* sp., respectively, for test complexes A, B, and C whereas diameter of zone of inhibition of Nystatin was found to be 18 mm, 28 mm, and 20 mm, respectively, against the organism. The antifungal activity (zone of inhibition) of the test complexes against respective fungi is presented in Table 13. The minimum inhibitory concentration (MIC) of the test complex A is 64 *μ*g/disc; for B and C it is 128 *μ*g/disc as listed in Table 14.

## 6. Antibacterial Activity of the Test Complexes

### 6.1. The Result of Antibacterial Activity of Test Compounds

The antibacterial activity of the test complexes was determined by using the dose of 80 *μ*g/disc. The results of antibacterial activity measured in terms of zone of inhibition are shown in Table 15. The complexes showed minimum sensitivity against the following number of both Gram-positive and Gram-negative bacteria and the results were compared with antibiotic disc of kanamycin.

The histopathological investigation on the tumour showed a retardation of tumour growth, increase in the narcotic and inflammatory area, and increased hair follicles. The Schiff complexes showed significant antimicrobial activity compared to control. We definitely say that the synthesized complexes possess cytotoxic properties. Toxicological studies revealed that the complexes are much more toxic to liver and kidney. They altered all biochemical parameters of rat blood. The exact mode of action of the complexes is unknown to us. Further investigation is appreciated to investigate detailed mechanism of action and their effect in serum electrolyte before any clinical use, especially for the effective doses.

## Figures and Tables

**Table 1:** Analytical data^a^ and physical properties.

Complex notation	Complex	Colour	Melting point (°C)	% M	% C	% H	% N	Molar conductance (Ohm^1^cm^2^mol^−1^)	Magnetic moment *M*_eff_ (B.M)
A	K[Cu(SB-1)(SCN)]	Yellowish	175–177		45.22 (45.18)	2.42 (2.39)	7.53 (7.50)	29	1.95

B	[Cu(SB-2)NN] NN = aminopyridine	Greenish	228–230	17.14 (17.52)	58.27 (58.60)	4.05 (4.50)	11.33 (11.10)	35	1.90

C	[Cu(SB-2)NN] NN = phenylenediamine	Greenish	160–162	16.52 (16.05)	59.27 (58.89)	4.42 (4.10)	10.92 (10.50)	42	2.0

^a^The values found are given in parenthesis.

**Table 2:** Selected IR spectral data of the complexes (band maxima, cm^−1^).

Compound	*ν*(C=N)	*ν*(NH_2_)	*ν*(CS)	*ν*(M-O)	*ν*(M-N)	*ν*(M-S-C=N)	Others
K[Cu(SB-1)(SCN)]	1605		750	535	415	340	*ν*(C=N) = 2100

[Cu(SB-2)NN] C_18_H_17_O_2_CuN_3_		3290 3150		537	295		*ν*(C=N) = 1555

[Cu(SB-2)NN] C_19_H_17_O_2_CuN_3_		3315 3232		535	285		

**Table 3:** Electronic spectral data of the complexes (band maxima in cm^−1^).

Compound	Band I	Band II	Band III
K[Cu(SB-1)(SCN)]	15,267	19,157	22,000

[Cu(SB-2)NN] C_18_H_17_O_2_CuN_3_	15,395	19,500	24,000

[Cu(SB-2)NN] C_19_H_17_O_2_CuN_3_	15,400	19,890	23,995

**Table 4:** The effect of test compounds and bleomycin on EAC cell growth inhibition.

Experiment	Drugs	Dose	Number of EAC cells/mouse on 5th day after tumour (EAC) cell inoculation	% of cell growth inhibition
EAC			(9.61 ± 1.63) × 10^7^	
EAC + bleomycin		0.3 mg/kg	(0.49 ± 0.77) × 10^7^	94.90
A	Synthetic	8 mg/kg	(0.46 ± 0.62) × 10^7^	95.21
B	Synthetic	10 mg/kg	(1.21 ± 0.32) × 10^7^	87.40
C	Synthetic	16 mg/kg	(1.01 ± 0.78) × 10^7^	89.49

A = K[Cu(SB-1)(SCN)], B = [Cu(SB-2)NN], NN = 2-aminopyridine, and C = [Cu(SB-2)(NN)], NN = 2-phenylenediamine.

**Table 5:** The effect of test compounds on survival time of EAC cell bearing mice.

Name of experiment	Drugs	Dose	Mean survival time	% of increase life span
Control (EAC bearing mice)			(21.37 ± 1.7)	
EAC + bleomycin		0.3 mg/kg	40 ± 1.2	87.17
A	Synthetic	8 mg/kg	29 ± 2.6	35.70
B	Synthetic	10 mg/kg	25 ± 1.4	16.98
C	Synthetic	16 mg/kg	26.14 ± 1.1	22.32

A = K[Cu(SB-1)(SCN)], B = [Cu(SB-2)NN], NN = 2-aminopyridine, and C = [Cu(SB-2)(NN)], NN = 2-phenylenediamine.

**Table 6:** The effect of test compounds on survival time of EAC cell bearing mice.

Days	Control (EAC)	Bleomycin (0.3 mg/kg i.p.)	DMSO 25 mg/kg	A 8 mg/kg	B 10 mg/kg	C 16 mg/kg
0	00	00	00	00	00	00
2	0.77 ± 0.37	0.57 ± 0.17	0.76 ± 0.38	0.55 ± 0.13	0.64 ± 0.19	0.60 ± 0.26
4	0.98 ± 0.43	0.75 ± 0.22	0.97 ± 0.63	0.72 ± 0.22	0.84 ± 0.24	0.82 ± 0.17
6	1.30 ± 0.27	1.10 ± 0.24	1.31 ± 0.33	1.06 ± 0.24	1.21 ± 0.16	1.16 ± 0.31
8	1.54 ± 0.32	1.24 ± 0.14	1.57 ± 0.23	1.14 ± 0.12	1.37 ± 0.16	1.31 ± 0.14
10	1.78 ± 0.18	1.44 ± 0.30	1.76 ± 0.16	1.29 ± 0.19	1.48 ± 0.26	1.43 ± 0.22
12	2.13 ± 0.17	1.63 ± 0.16	2.14 ± 0.17	1.47 ± 0.22	1.77 ± 0.18	1.71 ± 0.14
14	2.55 ± 0.67	1.80 ± 0.23	2.55 ± 0.63	1.73 ± 0.32	1.93 ± 0.34	1.84 ± 0.18
16	3.94 ± 0.55	2.05 ± 0.27	3.93 ± 0.53	1.94 ± 0.14	2.17 ± 0.12	2.10 ± 0.21
18	4.44 ± 0.43	2.20 ± 0.15	4.40 ± 0.42	2.13 ± 0.12	2.47 ± 0.15	2.29 ± 0.23
20	5.13 ± 0.63	2.43 ± 0.11	5.14 ± 0.62	2.36 ± 0.16	2.73	2.49 ± 0.44

A = K[Cu(SB-1)(SCN)], B = [Cu(SB-2)NN], NN = 2-aminopyridine, and C = [Cu(SB-2)(NN)], NN = 2-phenylenediamine.

**Table 7:** The effect of test compounds on hematological parameters in normal and tumour bearing mice.

Name of experiment	HB level g/dL	RBC cell/mL	WBC (TC) cell/mL	Lymphocyte %	Neutrophil %	Monocyte %
Normal mice	13.65 ± 0.4	(7.96 ± 0.57) × 10^9^	(6.35 ± 0.26) × 10^6^	71.25 ± 0.91	19.19 ± 0.28	8.93 ± 0.23
N + A	12.25 ± 0.17	(6.16 ± 0.16) × 10^9^	(10.76 ± 0.17) × 10^6^	75.00 ± 0.83	21.79 ± 0.38	3.10 ± 0.34
N + B	10.23 ± 0.13	(5.93 ± 0.22) × 10^9^	(16 ± 0.23) × 10^6^	72.10 ± 0.34	26.23 ± 0.24	1.73 ± 0.17
N + C	11.89 ± 0.26	(6.33 ± 0.19) × 10^9^	(14 ± 0.23) × 10^6^	73.47 ± 0.83	24.21 ± 0.16	2 ± 0.21
EAC (mice)	7.11 ± 0.23	(2.40 ± 0.10) × 10^9^	(25.63 ± 0.18) × 10^6^	43.36 ± 0.43	38.23 ± 0.37	10 ± 0.28
EAC + A	11.25 ± 0.23	(6.04 ± 0.21) × 10^9^	(9.19 ± 0.36) × 10^6^	60.25 ± 0.68	26.65 ± 0.18	8 ± 0.86
EAC + B	10.14 ± 0.18	(5.62 ± 0.27) × 10^9^	(14.82 ± 0.41) × 10^6^	51.87 ± 0.78	34.11 ± 0.22	6.12 ± 0.92
EAC + C	10.34 ± 0.10	(5.89 ± 0.29) × 10^9^	(12.67 ± 1.40) × 10^6^	57.68 ± 1.37	33.20 ± 2.8	8.10 ± 3.10

A = K[Cu(SB-1)(SCN)], B = [Cu(SB-2)NN], NN = 2-aminopyridine, and C = [Cu(SB-2)(NN)], NN = 2-phenylenediamine.

**Table 8:** The effect of test compounds on serum alkaline phosphatase activity.

Name of experiment	ALKP activity (*µ*mol of PNPP hydrolyzed/min/mL serum)	Name of experiment	ALKP activity (*µ*mol of PNPP hydrolyzed/min/mL serum)
EAC	(8.56 ± 0.31) × 10^−3^	Normal value	(28.33 ± 0.71) × 10^−3^
EAC + DMSO	(28.96 ± 0.21) × 10^−3^	DMSO	(29.13 ± 0.14) × 10^−3^
EAC + A	(22.11 ± 0.46) × 10^−3^	N + A	(38.33 ± 0.27) × 10^−3^
EAC + B	(16.93 ± 0.49) × 10^−3^	N + B	(35.11 ± 0.45) × 10^−3^
EAC + C	(19.88 ± 0.88) × 10^−3^	N + C	(32.79 ± 0.67) × 10^−3^

**Table 9:** The effect of test compounds on the enhancement of peritoneal cells and macrophages of life.

Name of the experiment	Dose mg/kg	Total peritoneal macrophage cells (×10^6^)/mouse Mean ± SEM	Total peritoneal cells (×10^6^)/mouse Mean ± SEM
Normal	Tap water	3.23 ± 2.0	23.76 ± 0.321
A	8	5.23 ± 0.40	25.42 ± 0.66
B	10	4.10 ± 0.62	25.93 ± 0.19
C	16	4.91 ± 0.52	26.71 ± 0.44

A = K[Cu(SB-1)(SCN)], B = [Cu(SB-2)NN], NN = 2-aminopyridine, and C = [Cu(SB-2)(NN)], NN = 2-phenylenediamine.

**Table 10:** The effect of test compounds on generation of MDA by lipid peroxidation in serum of normal mice.

Name of the experiment	mmol MDA/mL serum
Normal mice (control)	6.71 ± 0.32
A	11.43 ± 0.67
B	10.11 ± 0.20
C	10.94 ± 0.39

A = K[Cu(SB-1)(SCN)], B = [Cu(SB-2)NN], NN = 2-aminopyridine, and C = [Cu(SB-2)(NN)], NN = 2-phenylenediamine.

**Table 11:** Histopathological effect of test compounds.

Name of the experiment	Observation			
	Size	Number of lymphocytes	Necrotic area	Inflammatory area
EAC	↑↑↑	↓↓↓		
A	↓↓↓	↑↑↑	↑↑↑	↑↑↑
B	↓	↑	↑	↑
C	↓↓	↑↑	↑↑	↑↑
Bleomycin	↓↓↓	↑↑↑	↑↑↑	↑↑↑

A = K[Cu(SB-1)(SCN)], B = [Cu(SB-2)NN], NN = 2-aminopyridine, and C = [Cu(SB-2)(NN)], NN = 2-phenylenediamine.

↑ = increase, ↑↑ = moderately increase, and ↑↑↑ = greatly increase.

↓ = decrease, ↓↓ = moderately decrease, and ↓↓↓ = greatly decrease.

**Table 12:** Effect of test compounds on total protein in peritoneal fluid.

Name of the complex	Dose (mg/kg)	Total protein mg/mL, P.F., mean ± SEM
Control (EAC)	Tap water	174.73 ± 2.81
A	8	165.22 ± 2.93
B	10	167.33 ± 3.23
C	10	166.97 ± 3.20

A = K[Cu(SB-1)(SCN)], B = [Cu(SB-2)NN], NN = 2-aminopyridine, and C = [Cu(SB-2)(NN)], NN = 2-phenylenediamine.

**Table 13:** Zone of inhibition of antifungal activity of test complexes.

Test fungus	Diameter of zone of inhibition (mm) of test complexes			Nystatin (45 *µ*g/disc)
	A	B	C	
*Tinea pedis *	4	2	3	18
*Aspergillus niger *	8	23	10	28
*Coniothyrium* sp.	22	6	8	20

A = K[Cu(SB-1)(SCN)], B = [Cu(SB-2)NN], NN = 2-aminopyridine, and C = [Cu(SB-2)(NN)], NN = 2-phenylenediamine.

**Table 14:** The summary of the results of minimum inhibitory concentration (MIC) of test complexes.

Name of fungus	MIC of test complex (*µ*g/mL)		
	A	B	C
*Aspergillus niger *	64	128	128
*Coniothyrium* sp.	64	128	128
*Tinea pedis *	80	160	160

A = K[Cu(SB-1)(SCN)], B = [Cu(SB-2)NN], NN = 2-aminopyridine, and C = [Cu(SB-2)(NN)], NN = 2-phenylenediamine.

**Table 15:** Result of antibacterial activity of test compounds.

Test bacteria	Diameter of zone of inhibition (mm) of test complexes			Kanamycin (Ts/25 *µ*g/disc)
	A	B	C	
*Bacillus subtilis *	7	5	4	16
*Streptococcus-β-haemolytica *	13	9	7	25
*Escherichia coli *	9	11	7	17
*Sarcina lutea *	8	9	7	19
*Klebsiella *	11	14	7	17
*Shigella flexneri *	6	11	6	14
*Shigella boydii *	9	26	8	24
*Shigella dysenteriae *	12	10	9	14
*Pseudomonas aeruginosa *	12	7	10	13

A = K[Cu(SB-1)(SCN)], B = [Cu(SB-2)NN], NN = 2-aminopyridine, and C = [Cu(SB-2)(NN)], NN = 2-phenylenediamine.
